# Peripheral and Central Effects of Melatonin on Blood Pressure Regulation

**DOI:** 10.3390/ijms151017920

**Published:** 2014-10-08

**Authors:** Olga Pechanova, Ludovit Paulis, Fedor Simko

**Affiliations:** 1 Institute of Normal and Pathological Physiology and Centre of Excellence for Nitric Oxide Research, Slovak Academy of Sciences, Bratislava 81371, Slovak Republic; E-Mail: ludo@lfuk.sk; 2 Department of Animal Physiology and Ethology, Faculty of Natural Sciences, Comenius University, Bratislava 81371, Slovak Republic; 3 Department of Pathophysiology, Faculty of Medicine, Comenius University, Bratislava 81371, Slovak Republic; E-Mail: fedor.simko@fmed.uniba.sk; 4 The Third Clinic of Internal Medicine, Faculty of Medicine, Comenius University, Bratislava 81371, Slovak Republic; 5 Institute of Experimental Endocrinology, Slovak Academy of Sciences, Bratislava 81371, Slovak Republic

**Keywords:** melatonin, hypertension, central nervous system (CNS), MT1 and MT2 receptors, reactive oxygen species (ROS)

## Abstract

The pineal hormone, melatonin (*N*-acetyl-5-methoxytryptamine), shows potent receptor-dependent and -independent actions, which participate in blood pressure regulation. The antihypertensive effect of melatonin was demonstrated in experimental and clinical hypertension. Receptor-dependent effects are mediated predominantly through MT1 and MT2 G-protein coupled receptors. The pleiotropic receptor-independent effects of melatonin with a possible impact on blood pressure involve the reactive oxygen species (ROS) scavenging nature, activation and over-expression of several antioxidant enzymes or their protection from oxidative damage and the ability to increase the efficiency of the mitochondrial electron transport chain. Besides the interaction with the vascular system, this indolamine may exert part of its antihypertensive action through its interaction with the central nervous system (CNS). The imbalance between the sympathetic and parasympathetic vegetative system is an important pathophysiological disorder and therapeutic target in hypertension. Melatonin is protective in CNS on several different levels: It reduces free radical burden, improves endothelial dysfunction, reduces inflammation and shifts the balance between the sympathetic and parasympathetic system in favor of the parasympathetic system. The increased level of serum melatonin observed in some types of hypertension may be a counter-regulatory adaptive mechanism against the sympathetic overstimulation. Since melatonin acts favorably on different levels of hypertension, including organ protection and with minimal side effects, it could become regularly involved in the struggle against this widespread cardiovascular pathology.

## 1. Introduction

Essential hypertension is a complex hemodynamic and structural disorder, whose underlying mechanisms involve several familiar pathogenic factors. Genetic background, renal alteration, neurohumoral imbalance or endothelial dysfunction are generally accepted participating factors in hypertension development and progression. Besides peripheral alteration, also central nervous system disorders are involved. Thus, the term neurogenic hypertension was suggested, involving the imbalance between sympathetic and parasympathetic components on the base of the disturbed interplay on the level of the central and peripheral autonomic nervous system [1,2]. Recently, it has been suggested that the inflammation in the brainstem may underlie this neurogenic hemodynamic disorder [2]. Pro-inflammatory molecules, e.g., junctional adhesion molecules, are overexpressed in the endothelium of the microvasculature in the nucleus tractus solitarii, the principal structure controlling arterial blood pressure (BP), with a subsequent leukocyte adherence to inflamed endothelium and inflammatory cytokines release, while this type of inflammatory response seems to be quite specific for the hypertensive brainstem [3]. If the endothelium inflammation in the variable parts of the central nervous system is involved in the pathogenesis of hypertension in a more general way, then substances with a potential anti-inflammatory, antioxidant and endothelium-protecting action in the central nervous system (CNS), such as melatonin, might become important players in the therapeutic targeting.

Melatonin is an endogenous product of the pineal gland, acting as a messenger of the suprachiasmatic nucleus and synchronizing the daily rhythms of variable physiological functions [4,5,6,7,8]. Melatonin plays an important role in the regulation of several parameters of the cardiovascular system [9,10,11], including blood pressure, and is considered to be a putative antihypertensive agent [12,13,14]. However, the mechanisms and pathways involved in its BP lowering action are complex and not entirely clear. Both effects, mediated by specific melatonin receptors and direct unspecific actions, particularly those involving the antioxidant nature of melatonin, are of significant biological value. Melatonin and its metabolites [15,16] have extraordinary antioxidant potential and reduce the level of free radical burden on the level of both oxygen and nitrogen species [7,15,16,17], and their lipophilic action enables them to cross the cell membrane and extend the protective action to all subcellular structures [18,19,20]. Acting as a direct scavenger, melatonin is able to neutralize different free radicals, such as singlet oxygen, superoxide anion radical, hydroperoxide, hydroxyl radical, lipid peroxide radical and highly toxic peroxynitrite anion [21,22,23]. Indirect antioxidant actions of melatonin reside in the improvement of mitochondrial efficiency [24], stimulation of gene expressions and activation of superoxide dismutase (SOD), catalase and glutathione peroxidase [25]. Furthermore, the ability of melatonin to potentiate the antioxidant action of substances with an antioxidant potential, like glutathione, vitamin E and vitamin C, may also contribute to better vascular functions and blood pressure regulation [26,27,28].

## 2. Receptor-Dependent Effects of Melatonin on Blood Pressure

Melatonin receptors influence second messenger cascades that may vary in distinct cell, tissue and species. Specific melatonin receptors were described in the cellular membrane systems, cytosol and even nucleus. Both MT1 and MT2 are membrane-bound G protein-coupled receptors (GPCR). MT1 is primarily linked with Gαi and Gαq subunits, while MT2 is mainly connected with Gαi [29,30]. The cytosolic MT3 melatonin receptor is actually the quinone reductase 2A, having a low-affinity binding site [31,32]. This enzyme with a supposed effect on the cellular redox state was observed in mammals, but not yet found in humans. Melatonin may directly or indirectly (via MT1) modulate the activity of the nuclear retinoid orphan receptor/retinoid Z receptor (ROR/RZR), with possible indirect antioxidant effects [30].

The activation of Gαi reduces the intracellular level of cAMP, while Gαq stimulation results in phospholipase C activation, diacylglycerol and inositol-3-phosphate production and increased intracellular Ca^2+^ levels [33]. The activation of these second messenger cascades in the smooth muscle cells of the peripheral arteries corresponds with the melatonin-induced vasoconstriction observed in the rat caudal artery [34,35]. However, it is partly contradictory to the vasodilator effects of melatonin in the mesenteric artery and aorta [36,37]. It was hypothesized that the activation of melatonin receptors on endothelial cells would trigger nitric oxide (NO) formation, whose vasodilatory potential could overwhelm the MT-dependent contraction of smooth muscle cells [38]. Because this process is calcium-dependent, this hypothesis could also provide a rationale for the observed disappearance of the blood pressure reducing effects of melatonin in hypertensive patients controlled by nifedipine [39].

Another possible explanation for melatonin-mediated vasodilation may be Gαi-mediated activation of Kir 3 potassium channels [40]. Melatonin-related smooth muscle cell sarcolemma hyperpolarization might reduce the voltage-dependent channel opening and contribute to vasorelaxation [41].

The melatonin receptor subtype distribution in peripheral arteries can significantly influence the biological effect of melatonin. MT1 expression was observed in the rat aorta [42,43,44], while these receptors were surprisingly localized mainly in the tunica adventitia and not in the smooth muscle and/or endothelial cells [43], and in rat caudal artery [45]. MT2 were observed in porcine coronary arteries [46], in rat caudal artery [45], but not in the aorta [43]. The biological effect of MT1/MT2 stimulation in terms of vascular constriction or dilation may vary in dependence of the particular vessel or the condition of smooth muscle cells layer [45,47].

However, MT receptors may be involved in BP regulation also through the central regulatory mechanisms, since the highest density of melatonin receptors has been shown to be in the central nervous system, particularly in the adenohypophysis [48], suprachiasmatic nucleus (SCN) [49], paraventricular nucleus (PVN) [50] and area postrema [51]. Anyway, although MT1 and MT2 receptor-melatonin interactions may be of importance, they do not seem to be the major player in the blood pressure regulation by melatonin.

## 3. Non-Specific (Receptor-Independent) Actions of Melatonin in Blood Pressure Regulation

### 3.1. Melatonin in Ca^2+^ Metabolism

The antioxidative activities of melatonin are considered to be the principal receptor-independent effects of melatonin modulating the blood pressure level. Melatonin’s lipophilic nature enables this indolamine to cross the cell membrane and reach subcellular components, including nucleus [52]. The Ca^2+^-calmodulin complex plays a major role among the intracellular melatonin targets. Nanomolar concentrations of melatonin interact with the Ca^2+^-calmodulin complex, modifying thusly its effects in many physiological and pathophysiological conditions [53].

The Ca^2+^-calmodulin interactions with melatonin in the vascular bed result in the modification of intracellular Ca^2+^ concentrations. While in smooth muscle cells, the melatonin effect on the Ca^2+^-calmodulin complex may decrease the level of Ca^2+^ and lead to relaxation, in endothelial cells, the reduced Ca^2+^ level may inhibit endothelial nitric oxide synthase (NOS), triggering thusly vasoconstriction [54]. Suggestively, the biological effect of the melatonin-Ca^2+^-calmodulin interplay depends on the type of target cell.

### 3.2. Melatonin in the Reduction of Free Radical Burden

Receptor-independent functions have mostly been discussed in relation to the antioxidant and overall redox-dependent effects of melatonin in experimental models of hypertension [38,55,56,57,58,59] and hypertonics [13,60]. The antioxidant nature of melatonin involves both direct scavenging of reactive oxygen species (ROS) exerted by high-millimolar concentrations of melatonin or stimulation of the activity and expression of antioxidant enzymes, a response observed at low nanomolar concentrations. Regarding scavenging actions of this indolamine, a potential product of the melatonin interaction with OH has been identified as cyclic 3-hydroxymelatonin [61,62] with H_2_O_2_ as *N*^1^-acetyl-*N*^2^-formyl-5-methoxykynuramine (AFMK) [63], while both 3-hydroxymelatonin and AFMK are effective antioxidants themselves [16,64].

Increased activity and expression of different antioxidant enzymes by melatonin were extensively documented. Long-term melatonin administration (two months) increased catalase activity, leading to the reduction of oxidative stress parameters along with attenuation of hypertension in patients with metabolic syndrome [60], and melatonin administration for 15 days increased SOD-1 activity in elderly hypertonics [65]. It was suggested that melatonin modulates antioxidant enzyme activities via its interaction with calmodulin, which, in turn, inhibits downstream processes inactivating nuclear RORα receptor, the member of the nuclear (orphan) receptor family, which downregulates NF-κB-induced antioxidant enzyme expression [25].

In spontaneously hypertensive rats, melatonin decreased the NF-κB (p65) expression [55], while the inhibition of NF-κB pathway in rat endothelial cells reduced lipopolysaccharide-induced NO production [66]. Melatonin seems to interact differentially with the NF-κB pathway, depending supposedly on the redox and/or inflammatory state in the particular cardiovascular pathology, including hypertension.

## 4. Melatonin and Sympathetic Activity

The physiology of melatonin is closely bound with the sympathetic nervous system. On the one hand, the melatonin release is controlled by sympathetic afferentation to the pineal gland, mediating the inhibitory effect of light on pineal melatonin secretion [67]. This pathway starts in the retina, influencing the master biological clock in the SCN [68,69]. The SCN then inhibits the PVN by γ-aminobutyric acid (GABA)-ergic innervation [68], leading to interruption of the constant stimulation of the sympathetic intermediolateral nucleus by the PVN [70]. This sympathetic pathway (including interpolation in the superior cervical ganglion) induces the production of melatonin [68] by stimulation of pineal β1- and α1-adrenoceptors [71,72,73] in rodents or primates, although the actual intracellular mechanism of melatonin production modulation may involve several different ways [74]. Interestingly, the production of melatonin is followed by reduced NF-κB translocation to the nucleus [75].

On the other hand, melatonin modulates the tone of the autonomic nervous system. Pinealectomized rats showed higher catecholamine levels upon interleukin-1-β stimulation, an effect that was abolished by intraventricular infusion of melatonin [76]. In spontaneously hypertensive rats (SHR), acute administration of melatonin reduced blood pressure along with norepinephrine levels [77]. Acute administration of melatonin to normotensive rats reduced blood pressure and heart rate, along with the reduction of serotonin levels in the corpus striatum and hypothalamus [78]. Moreover, in these experiments, the effect of melatonin on blood pressure and heart rate was abolished by spinal transection or bilateral vagotomy, suggesting the involvement of the sympathetic inhibition or parasympathetic stimulation by melatonin [78]. Chronic administration of melatonin (similarly to the antioxidant *N*-acetylcysteine) decreased the blood pressure and heart rate, improved the chronotropic response to isoproterenol, in association with an inhibition of sympathetic activity and the restoration of cardiac β-adrenoceptor function [79] and improvement of baroreflex [80]. In healthy young men, melatonin reduced the pulsatile index and systolic blood pressure along with norepinephrine levels [81] and reduced blood pressure and pulse wave velocity in association with the attenuation of sympathetic tone [82]. These sympatholytic effects of melatonin may be involved in the blood pressure reducing effect of melatonin seen in SHR [55], N^G^-nitro-l-arginine methyl ester (l-NAME) hypertensive rats [56,57], healthy volunteers [81] or patients with essential hypertension [13,29,83,84,], and it also participates in the improvement of insomnia and depression [85].

## 5. Melatonin Effects in Central Blood Pressure Regulation

### 5.1. Melatonin Modulation of Central Nervous System (CNS) Sites

The partly contradictory effects of melatonin on vascular reactivity [38,86], the association of melatonin administration with sympatholytic effects [79,87] and the dependence of melatonin-induced blood pressure decrease on intact spinal cord [78], all suggest a prominent role of the central effects of melatonin on blood pressure regulation. The precise site of this action, however, is still not unequivocally elucidated. Previously, several possible sites for the modulation of central nervous system output by melatonin were suggested [38]:

- The activity of the SCN might be modulated by melatonin activity [69,88], reducing thusly the sympathetic tone and providing a protective mechanism against excessive sympathetic excitation.
- In neurons projecting from the SCN to the PVN [70] or in neurons projecting from the caudal ventrolateral medulla (CVLM) to the rostral ventrolateral medulla (RVLM) [89], the GABA-ergic signaling might be potentiated by melatonin [90], either directly or via enhancement of the NO bioavailability [91].
- In the area postrema, which inhibits the activity of RVLM through CVLM [89], melatonin is supposed to modify the epigenetic effect [92].

In addition, in all of these brain targets, melatonin prominent antioxidative nature may participate in the attenuation of the sympathetic tone. Moreover, it has been shown that in stress-induced hypertensive rats, melatonin levels in the anterior hypothalamic area were reduced. A microinjection of melatonin into this site reduced blood pressure along with increased GABA-ergic activity and reduced glutamate-ergic activity in the RVLM. This effect was prevented by MT1/MT2 blockade [93]. Moreover, in this model of hypertension, also chronic intraperitoneal administration of melatonin prevented the increase in blood pressure, whose effect was sensitive to a GABA receptor blockade [94]. On the other hand, the deletion of the SCN abolished circadian blood pressure rhythms, but did not affect absolute blood pressure levels in rats with over-expression of renin [95]. Moreover, periodical administration of melatonin (during the dark phase) did not affect the expression of clock genes or melatonin receptors in the SCN of these rats [96]. It could be suggested that while the action of melatonin on the SCN might interfere with the nocturnal blood pressure level, the effect of melatonin on RVLM (most likely associated with augmented GABA-ergic signaling) may inhibit the sympathetic tone and induce the overall blood pressure reduction independently of the light-dark daily periods.

### 5.2. Melatonin, Oxidative Stress and Angiotensin II Interactions

It is generally admitted that peripheral mechanisms of BP regulation and hypertension treatment are substantially better understood than those at the level of CNS, involving predominantly the relative dominance of sympathetic and renin-angiotensin systems.

Angiotensin II represents a key factor enhancing ROS production in the central nervous system, predominantly by the activation of nicotinamide adenine dinucleotide phosphate (NADPH) oxidase. Besides the cardiovascular system, also the CNS suffers seriously from the long-term impact of the increased reactive oxygen or nitoroso species. The brain is remarkably susceptible to the oxidative stress, because its antioxidant defense is rather poor [28]. In the brain, only the low catalase activity and moderate levels of the antioxidant enzymes, like superoxide dismutase and glutathione peroxidase, were detected. The high levels of iron and ascorbic acid in the brain participate significantly in the catalysis of lipid peroxidation. Additionally, neurotransmitters may be autoxidized, generating thusly ROS [97]. Thus, the antioxidant, scavenging and anti-inflammatory effects of melatonin in the CNS may additionally contribute to blood pressure reduction. Melatonin is able to increase the activity and/or mRNA of glutathione peroxidase, copper-zinc superoxide dismutase, manganese superoxide dismutase and reduced glutathione in different brain regions, observed during both acute and chronic treatments with melatonin [25,98]. Furthermore, melatonin directly affects the assembly of NADPH oxidase in microglia, potentially through the inhibition of NADPH oxidase phosphorylation via a PI3K/Akt-dependent signaling pathway, blockade of p47(phox) and p67(phox) subunits translocation to the membrane and downregulation of p47(phox) binding to gp91(phox) [99].

Importantly, angiotensin II itself increases melatonin synthesis in the pineal gland [100,101], which may be considered as the self-defending mechanisms restraining the deleterious effects of chronic angiotensin II activation, including hypertension and pathologic remodeling development.

### 5.3. Melatonin and Nitric Oxide Interplay

The relation of melatonin regarding its effect on nitric oxide level is rather complex in the brain tissue. This indolamine and its derivates inhibit neuronal NOS (nNOS) and inducible NOS (iNOS), while the effect on endothelial NOS (eNOS) is less clear. Melatonin inhibits iNOS through the NF-κB-dependent signaling pathway [102,103,104] and eNOS via modification of the Ca^2+^-calmodulin complex [105]. nNOS is activated by calcium similarly to eNOS, and an analogic mechanism in the melatonin-nNOS interaction in the brain may be supposed. The study of [106], however, documented that melatonin prevented a decrease of eNOS expression during ischemic brain injury. Thus, it is plausible that the scavenging and antioxidant effect of melatonin may stabilize eNOS and potentially also the nNOS isoform. It seems that the final melatonin effect on different NOS isoforms may vary according to the pathophysiological conditions, dose or strain tested [107]. In any case, the melatonin-nitric oxide pathway interplay at the level of the CNS does not seem to be the crucial factor in BP regulation.

## 6. The Potential Role of Melatonin in Nocturnal Blood Pressure Regulation

Several lines of evidence indicate that disturbed BP rhythmicity-chronodisruption with reduced melatonin secretion is involved in the pathophysiology of nocturnal BP alterations [108].

First, blood pressure undergoes typical circadian rhythmicity with a steep increase in the morning, higher values throughout the active walking period and a deeper decline during nocturnal rest (10% to 20% decline of daily values) [109]. Subjects with attenuated BP decline, non-dippers or those with nocturnal hypertension have increased risk of cardiovascular events compared to the common population [110], and nocturnal blood pressure has even been identified as a better predictor of cardiovascular outcome than daily BP values [111]. Most importantly, the shifting of antihypertensive therapy to evening hours not only improved BP control and reduced the number of non-dippers, but remarkably improved the prognosis during the long term, followed up in the MAPEC (Monitorización Ambulatoria para Predicción de Eventos Cardiovasculares/Ambulatory Blood Pressure Monitoring and Events) study [112]. Thus, the chronodisruption of normal BP rhythmicity seems to play a significant role in the prognosis and is an important therapeutic target.

Secondly, it is generally known that under physiological conditions, the level of melatonin is high during night and is inhibited by daily light to very low levels [9,108]. The evening rise in melatonin represents an important part of suprachiasmatic-induced adaptation to the rest and activity period [113]. On the other hand, non-dipping patients express a lower nocturnal surge of melatonin release, as reflected by reduced 6-sulfametoxymelatonin in urine [114] and a lower ratio of the night/day concentration of melatonin [115]. Furthermore, experimental pinealectomy in rats reduced both nocturnal and daily melatonin levels [116], as well as the urinary excretion of 6-sulfatoxymelatonin in rats [117]. Additionally, no nocturnal rise in melatonin secretion was observed in pinealectomized patients [118]. Analogically, exposing hamsters to a longer period of continuous light reduced amplitude and shortened duration of enhanced melatonin concentration in the pineal gland and attenuated nocturnal excretion of aMT6s into the urine [119]. This reduction of melatonin secretion was associated with hypertension development in laboratory animals [20].

Thirdly, in the agreement with the proven melatonin deficiency in certain types of hypertension, the supplementation of pinealectomized or continuous light-exposed animals with melatonin prevented hypertension development [120]. In the clinical setting, several weeks of melatonin administration before sleep reduced nocturnal systolic and diastolic BP in non-dipping untreated patients [113], in non-dipping women [87] and in patients with nocturnal hypertension [121]. In agreement with physiological enhancement of melatonin secretion during the night, melatonin should be applied at bedtime. Daytime melatonin intake results in sleepiness and hypothermia during the day [122] and should be avoided [113].

Fourthly, the patho-mechanism of the BP reducing effect of melatonin seems to be unrelated to the well-known effect of melatonin on the quality of sleep. Repeated melatonin intake significantly increases sleep deficiency and actual sleep time and reduces sleep latency. However, although three-week night-time melatonin administration to untreated non-dippers resulted in lowering nigh-time BP levels along with the improvement of sleep, improvement in hemodynamics and sleep were statistically unrelated [113]. Similarly, in young patients with diabetes type 1, melatonin significantly reduced diastolic BP; however, no effect on sleep duration or sleep quality was observed [122].

## 7. Questions and Perspectives

Despite the vast evidence of melatonin’s ability to reduce BP and its potential role in BP regulation at the peripheral and central levels, the causative relation of melatonin to the pathogenesis of hypertension development remains controversial. This uncertainty is associated with the variable findings regarding the plasmatic or tissue melatonin profile during hypertension, which may prove to be decreased, normal or even increased.

There is accumulating evidence that the function of the circadian pacemaker in the SCN is deteriorated in essential hypertension. Hypertensive patients show blunted day-night rhythms in sympathetic and parasympathetic heart tone [123]. The levels of neurotransmitters in SCN are significantly reduced, and the SCN output to the sympathetic nervous system is altered in patients with hypertension [124,125]. These changes in the vegetative nervous system may be expected to induce some counter-regulatory mechanisms. Indeed, in untreated hypertensive patients, the morning concentrations of melatonin were higher compared to normotensives, and the level of melatonin was normalized after blood pressure reduction by six-month lacidipine treatment [126]. It has been shown in our laboratory that melatonin level in the pineal gland was increased in l-NAME–induced hypertension compared to controls. It was suggested that increased sympathetic tone or reduced NO formation in the PVN in l-NAME-treated rats might have underlain the increased (potentially compensatory) melatonin production. However, in the same experiment, in peripheral organs, melatonin levels were not substantially changed. It has been suggested that increased melatonin production was insufficient to counterbalance the augmented melatonin consumption in the peripheral tissues [42]. Similarly, in rats with chronic immobilization stress and increased sympathetic nerve activity and enhanced circulating norepinephrine levels, the nocturnal melatonin levels were increased [127].

## 8. Conclusions

Although melatonin reduces blood pressure and its deficit seems to be one of the substantial factors in the pathogenesis of hypertension, its production or serum level does not need to be unavoidably reduced in the condition of increased BP. On the contrary, melatonin production can be enhanced along with the augmented sympathetic tone triggering melatonin release, at least in some forms of hypertension. It seems, however, that this supposedly compensatory increase of endogenous melatonin production fails to provide sufficient counter-regulation to the activated autonomic nervous system and can thus be viewed as a “relative” melatonin deficiency.

It is exciting to suppose that artificial light pollution, a general phenomenon of the urbanized world, may be a factor of melatonin level alteration and a risk factor for hypertension development. If inadequate melatonin production may be causally associated with increased nocturnal or even daily BP, improvement of chronodisruption may be a hopeful therapeutic approach. It does not seem unreasonable to suppose that the correction of absolute or relative melatonin deficiency by exogenous melatonin administration may help to attenuate the excessive catecholamine outflow, providing a rational background for therapeutic application of melatonin in hypertension treatment.
